# 

**Published:** 1892-07

**Authors:** 


### CONTENTS.

### PROCEEDINGS.

Eighteenth Annual Session of the Mississippi Dental Association... 307
Post-Graduate Dental Association ........................................ 348

### COMMUNICATIONS.

The Diagnosis of Chancre—Prognosis of Treatment........................ 328
Some of the Results of the Early Extraction of the Sixth Year Molar ........................................................................ 334
The Battle with Germs.............................................................. 339
A Dental Law in the District of Columbia.................................. 345

### NOTICES OF DENTAL MEETINGS.

Pennsylvania and New Jersey State Societies.............................. 351
National Association of Dental Faculties .................................. 351
American Dental Association...................................................... 351
South Carolina State Dental Association..................................... 352
California State Dental Association .......................................... 352
National Association of Dental Examiners ................................. 352

### EDITORIAL.

About Dental Colleges................................................................. 353
World's Columbian Dental Congress ........................................... 357
The Southern Dental Congress................................................... 358
American Dental Association...................................................... 360

